# Pre-operative Labs for Left Hemicolectomy Reveals Hyponatremia That Leads to Lung Mass Finding

**DOI:** 10.7759/cureus.43865

**Published:** 2023-08-21

**Authors:** Jeffrey A Tyre, Patrick Narh-Martey, Natalee M Tyre

**Affiliations:** 1 Medicine, Trinity School of Medicine, Warner Robins, USA; 2 General Surgery, Houston Healthcare, Warner Robins, USA

**Keywords:** siadh, copd, small cell lung cancer, hyponatremia, paraneoplastic syndrome

## Abstract

Paraneoplastic syndromes are a group of clinical conditions with specific signs and symptoms that are associated with underlying malignancies. The pathophysiology of paraneoplastic syndromes is caused by either the ectopic production of various hormones or the immune production of autoantibodies. Lung cancers are also notorious for being accompanied by paraneoplastic syndromes. The endocrine paraneoplastic syndromes most commonly associated with lung cancer include hypercalcemia of malignancy and syndrome of inappropriate antidiuretic hormone secretion (SIADH). Oftentimes, one of the initial findings in the early stages of lung malignancy is not symptoms of a primary lung neoplasm, but the symptoms and/or lab findings of a paraneoplastic syndrome.

This article has been written to present a case of how an asymptomatic patient with a lung mass and lab values suggesting SIADH could benefit from an alteration to the current status quo in the work-up of hypo-osmolar hyponatremia.

The main reason for writing the article is to suggest incorporating one of the United States Preventive Services Task Force (USPSTF) guidelines into the current work-up of hypo-osmolar hyponatremia. Currently, the workup for hypo-osmolar hyponatremia says, “consider chest imaging.” However, incorporating one of the USPSTF screenings into a questionnaire for medical providers could be beneficial in identifying lung abnormalities sooner in patients with a smoking history and also be more appropriate in determining whether a patient should receive chest imaging.

## Introduction

Paraneoplastic syndromes of neuroendocrine origin are often associated with lung malignancy, especially small-cell lung cancer (SCLC). These paraneoplastic syndromes commonly present before evidence of the underlying malignancy [1,2]. The paraneoplastic syndrome associated with SCLC is SIADH. Squamous cell carcinoma of the lung is associated with ectopic production of parathyroid hormone-related peptide (PTHrP), which causes hypercalcemia and hyperphosphatemia. Therefore, patients with hyponatremia of unknown etiology or hypercalcemia and hyperphosphatemia with a history of cigarette smoking should be evaluated for lung malignancy. The patient presented with hyponatremia, low BMI, and an extensive history of tobacco smoking. He was later found to have multiple lung abnormalities on a CT scan.

## Case presentation

**Figure 1:**
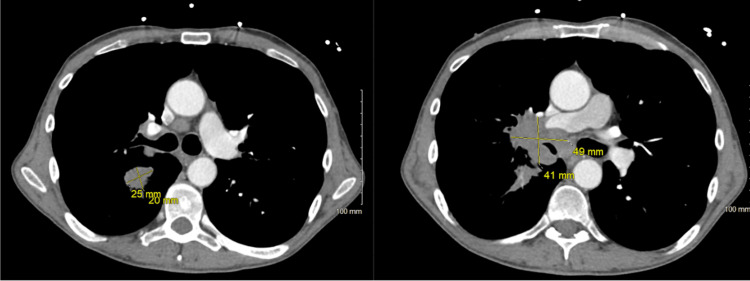
Chest CT revealing two images at two separate planes showing two different right-sided lung masses.

A 58-year-old male was admitted to the hospital after his pre-operative labs for a left hemicolectomy that revealed asymptomatic, severe hyponatremia. The left hemicolectomy had been scheduled due to adverse findings on colonoscopy. The patient appeared cachexic with a BMI of 17.2 and had been a cigarette smoker since he was eight years old. There was no tenderness or guarding to palpation of the abdomen. The lungs were clear to auscultation bilaterally. Overall, the patient’s physical exam was unremarkable except for his cachectic appearance. On admission, vital signs were within normal limits except for the oxygen saturation ranging from 92% to 94%. Initial sodium (120 mEq/L), chloride (90 mEq/L), and calculated osmolality (240.2 mOsm/kg) levels were found to be decreased. Urine osmolality was 662 mOsm and urine sodium was 121 mEq/L. Blood coagulation studies were unremarkable. His pre-operative American Society of Anesthesiologists score for surgical clearance was two. When sodium levels were corrected with medical intervention, the patient underwent an uncomplicated and successful left hemicolectomy with anastomosis. The pathology report of the left colon was negative for invasive carcinoma but did identify a non-invasive tubulovillous adenoma. CT scan of the chest was then performed due to the existence of continued decreased sodium levels after the potential malignancy in the colon had been removed. Current guidelines say to consider chest imaging, so chest imaging was put off until after the colon surgery due to no pulmonary symptoms, and there was a reasonable chance the paraneoplastic syndrome was coming from the possible malignancy in the colon. CT scan of the chest (Figure 1) was performed post-operatively and revealed the following findings: right hilar mass measuring 5 cm x 4 cm, bilateral nodularities in the lower lobes w/bilateral atelectasis, a spiculated nodule in the right lower lobe measuring 2.5 cm x 2 cm, and diffuse emphysematous change. The liver was unremarkable on CT imaging. The patient signed out against medical advice before he was given the results of the CT scan. Therefore, a lung biopsy was not obtained to confirm the presence of paraneoplastic syndrome. The patient was notified of the chest CT abnormalities by the surgeon at a follow-up visit and was referred to a primary care physician for further evaluation.

## Discussion

**Figure 2:**
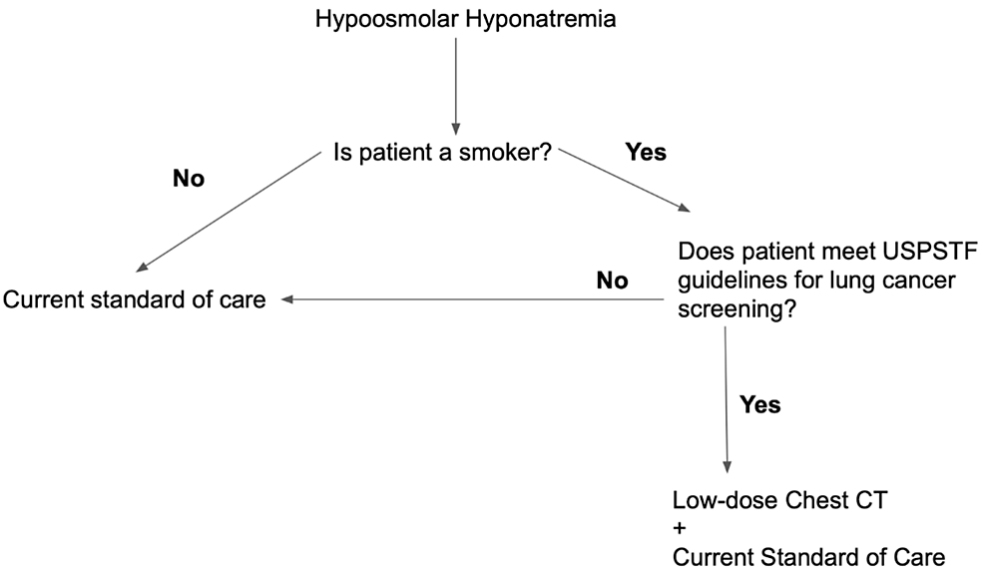
Suggested improvement to the current guidelines in regard to hypo-osmolar hyponatremia. USPSTF, United States Preventive Services Task Force

The patient presented with asymptomatic hyponatremia, low BMI, and an extensive history of tobacco smoking. The patient had no concerning respiratory symptoms. He was later found to have multiple lung abnormalities on CT scan. The patient’s presentation with hyponatremia and lung findings on CT suggest a primary SCLC given the association with SIADH without invasion of the dysplasia of the colon biopsy and having an unremarkable liver on CT scan. It is important to identify SCLC in its early development because 70% of SCLC present with metastatic disease [3]. The current United States Preventive Services Task Force (USPSTF) recommends lung cancer screening in adults aged 50 to 80 years old who have a 20-pack-year smoking history and who currently smoke or have quit smoking cigarettes in the past 15 years. However, preventative screening is usually performed by a primary care physician. The patient in this report does not have a primary care physician and thus never had the necessary lung screening even though he is in the category that should have been screened. Since he was found to have SIADH, a work-up needs to be done to rule out malignancy or other causes of SIADH. Delayed identification of a paraneoplastic syndrome and subsequent treatment of lung cancer [4]. The algorithm could use a mandate to incorporate current screening guidelines to improve the quality of care in the management of potential paraneoplastic syndrome (Figure 2), especially since the current algorithm suggests chest imaging in patients with hypo-osmolar hyponatremia without further instructions on who specifically to screen [5].

## Conclusions

Lung cancer is often found in the later stages of the disease. Incorporation of specific USPSTF guideline questionnaires in the work-up of hyponatremia and other findings of the paraneoplastic syndrome could potentially increase the early detection of lung cancer. For example, in a patient with hyponatremia, the physician should identify if the patient is a tobacco smoker and if the patient meets the criteria for lung cancer screening by USPSTF guidelines. This incorporation would benefit many patients who do not have access to primary care physicians. Other potential benefits of early detection of the etiology causing hyponatremia include a decreased hospital stay and reduced financial and resource burden on the healthcare system. The proposed changes to the guidelines for in-patient care have the potential to be significantly beneficial for both patients and the healthcare system alike.

## References

[1] Soomro Z, Youssef M, Yust-Katz S, Jalali A, Patel AJ, Mandel J (2020). Paraneoplastic syndromes in small cell lung cancer. J Thorac Dis.

[2] Yeung SC, Habra MA, Thosani SN (2011). Lung cancer-induced paraneoplastic syndromes. Curr Opin Pulm Med.

[3] Nikoomanesh K, Choi J, Arabian S (2018). Paraneoplastic syndrome as the presentation of limited stage small cell carcinoma. BMC Pulm Med.

[4] Hoorn EJ, Zietse R (2017). Diagnosis and treatment of hyponatremia: compilation of the guidelines. J Am Soc Nephrol.

[5] Goh KP (2004). Management of hyponatremia. Am Fam Physician.

